# In vivo imaging of the lung inflammatory response to *Pseudomonas aeruginosa* and its modulation by azithromycin

**DOI:** 10.1186/s12967-015-0615-9

**Published:** 2015-08-04

**Authors:** Fabio Stellari, Gabriella Bergamini, Angela Sandri, Gaetano Donofrio, Claudio Sorio, Francesca Ruscitti, Gino Villetti, Barouk M Assael, Paola Melotti, Maria M Lleo

**Affiliations:** Pharmacology and Toxicology Department Corporate Pre-Clinical R&D, Chiesi Farmaceutici S.p.A. Parma, Largo Belloli, 11/A, 43122 Parma, Italy; Dipartimento di Patologia e Diagnostica, Università di Verona, Verona, Italy; Dipartimento di Scienze Medico Veterinarie, Università di Parma, Parma, Italy; Dipartimento di Scienze Biomediche, Biotecnologiche e Traslazionali, Università di Parma, Parma, Italy; Centro Regionale Fibrosi Cistica, AOUI Verona, Verona, Italy

**Keywords:** In vivo bioluminescence imaging, Lung inflammation mouse model, *Pseudomonas aeruginosa*, Azithromycin

## Abstract

**Background:**

Chronic inflammation of the airways is a central component in lung diseases and is frequently associated with bacterial infections. Monitoring the pro-inflammatory capability of bacterial virulence factors in vivo is challenging and usually requires invasive methods.

**Methods:**

Lung inflammation was induced using the culture supernatants from two *Pseudomonas aeruginosa* clinical strains, VR1 and VR2, isolated from patients affected by cystic fibrosis and showing different phenotypes in terms of motility, colony characteristics and biofilm production as well as pyoverdine and pyocyanine release. More interesting, the strains differ also for the presence in supernatants of metalloproteases, a family of virulence factors with known pro-inflammatory activity. We have evaluated the benefit of using a mouse model, transiently expressing the luciferase reporter gene under the control of an heterologous IL-8 bovine promoter, to detect and monitoring lung inflammation.

**Results:**

In vivo imaging indicated that VR1 strain, releasing in its culture supernatant metalloproteases and other virulence factors, induced lung inflammation while the VR2 strain presented with a severely reduced pro-inflammatory activity. The bioluminescence signal was detectable from 4 to 48 h after supernatant instillation. The animal model was also used to test the anti-inflammatory activity of azithromycin (AZM), an antibiotic with demonstrated inhibitory effect on the synthesis of bacterial exoproducts. The inflammation signal in mice was in fact significantly reduced when bacteria grew in the presence of a sub-lethal dose of AZM causing inhibition of the synthesis of metalloproteases and other bacterial elements. The in vivo data were further supported by quantification of immune cells and cytokine expression in mouse broncho-alveolar lavage samples.

**Conclusions:**

This experimental animal model is based on the transient transduction of the bovine IL-8 promoter, a gene representing a major player during inflammation, essential for leukocytes recruitment to the inflamed tissue. It appears to be an appropriate molecular read-out for monitoring the activation of inflammatory pathways caused by bacterial virulence factors. The data presented indicate that the model is suitable to functionally monitor in real time the lung inflammatory response facilitating the identification of bacterial factors with pro-inflammatory activity and the evaluation of the anti-inflammatory activity of old and new molecules for therapeutic use.

## Background

Airway inflammation is a central component of a number of chronic lung diseases such as asthma, chronic obstructive pulmonary disease (COPD), cystic fibrosis (CF) and bronchiectasis. Airway inflammation is characterized by edema, cellular infiltration, activated T lymphocytes and mast cells, increased airway secretions, and deposition of excess collagen [1, 2]. Frequently, the inflammation is associated with bacterial infections such as those caused by *Pseudomonas aeruginosa*, an opportunistic human pathogen involved in severe airway infections especially in patients suffering from CF and COPD [1, 3]. During the early onset of the lung infection, *P. aeruginosa* secretes a high number of virulence factors which are responsible for tissue damage and inflammation [4]. As the infection progresses, the bacterium switches off most of the virulence genes but synthesizes a biofilm matrix and becomes resistant to antibiotics causing a chronic disease frequently leading to respiratory failure and lung transplantation or death [4].

Therefore, it is mandatory to identify those factors and conditions causing lung cell damage and favoring the passage from an acute to a chronic bacterial infection by monitoring, for long times, the inflammation process. Furthermore, to avoid the onset of the chronic phase of the infection, it is important to treat *P. aeruginosa* infection during the acute phase using efficient antibiotic therapy and anti-inflammatory drugs.

By standard methods, the inflammation of the respiratory tract can be monitored by counting immunological markers recruited during the inflammatory process with sputum collection, a technique which provides poorly reliable results, or invasive sampling techniques such as bronchoscopy [5]. Animal models of acute and chronic lung infection have been used to study the bacterial behavior and for monitoring the host response in vivo [1, 6]. These models provide an important resource to identify essential bacterial genes for in vivo infection persistence and for the development and testing of new therapies [6, 7]. Recently, a mouse model, transiently expressing the luciferase reporter gene under the control of the bovine IL-8 promoter, has been described [8] and demonstrated to be suitable to functionally monitor in real time the lung inflammatory response [8–11]. This small size experimental animal model is based on the transient transduction of the IL-8 promoter, a gene representing a major player during inflammation, essential for leukocytes recruitment to the inflamed tissue and an appropriate molecular read-out for monitoring the activation of inflammatory pathways [8]. Although mice do not have an IL-8 (bIL-8) gene, mouse cell signaling and their transcriptional apparatus could specifically activate the bovine IL-8 gene promoter. Since lung disease manifestation in ruminants overlap with the majority of human lung disease manifestations, this model could be of great value to study human lung diseases too.

It has been shown that the *P. aeruginosa* strains isolated during the early phase of lung colonization had a pro-inflammatory capability higher than that induced by strains isolated during lung chronic colonization [12]. The pro-inflammatory effect, associated with the expression of IL-8 mRNA in CF airway epithelial cells, was shown to be associated to several *Pseudomonas* released proteins with proteolytic activity including members of the metalloprotease (MP) family [12]. Other virulence factors secreted by *Pseudomonas* such as exotoxins and exoenzymes, pyoverdine and pyocianine are also involved in tissue damage and inflammation. It has been suggested that azithromycin (AZM), an antibiotic used in CF patients, could elicit its anti-inflammatory activity by decreasing the synthesis of *Pseudomonas* exoproducts [13–15]. In fact, Molinari et al. [13] have shown that sub-lethal doses of AZM strongly suppressed the synthesis of elastase, lecithinase, proteases and DNase, while in another study it has been shown that AZM reduced the production of bacteria virulence factors by inhibiting quorum-sensing [15].

In the present study, we used the IL-8/luciferase transgenic mouse model for the in vivo monitoring of the IL-8 mediated lung inflammation induced by *P. aeruginosa* secreted pro-inflammatory virulence factors and their modulation by AZM.

## Methods

### Collection of bacterial cell-free supernatants

**Fig. 1:**
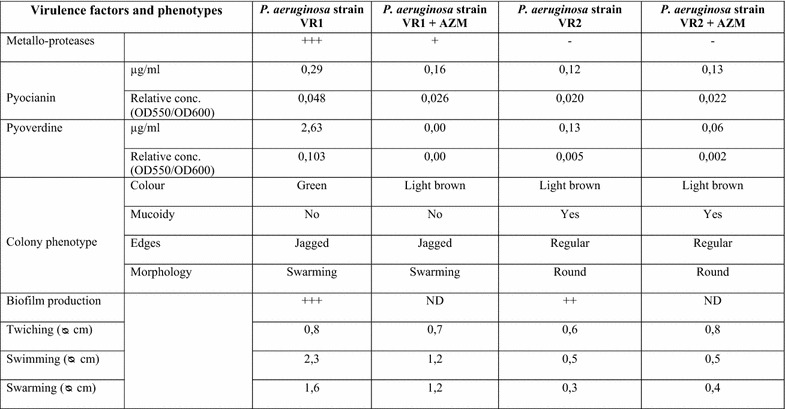
Phenotypic characteristics and virulence factors of the *P. aeruginosa* clinical strains.

Two *P. aeruginosa* strains, VR1 and VR2, were isolated from sputum samples of CF patients followed at the Cystic Fibrosis Center in Verona, Italy and were characterized for their differential expression of known virulence factors (data summarized in Fig. 1). Written informed consent was obtained from the subjects enrolled in the study and approved by the Institutional Review Board of Azienda Ospedaliera Universitaria Integrata (AOUI) Verona as project 1612.

Bacterial strains were grown overnight at 37°C in Bacto™ Tryptic Soy Broth (TSB, Becton, Dickinson and Company) with continuous agitation. The day after, *P. aeruginosa* cells were diluted in TSB to the concentration of 1 × 10^8^ CFU/mL (OD of 0.1 at 600 nm) and incubated in absence and in presence of 8 µg/mL of AZM, at 37°C for 16 h with continuous agitation. The concentration chosen for this antibiotic is in the sub-minimum inhibitory concentration (sub-MIC) range for *P. aeruginosa*, and is consistent with the concentrations found in lungs of patients treated with this drug [16]. By adding TSB, the cultures were normalized to an optical density of 0.2 OD at 600 nm. Culture supernatants (Sn) were collected by centrifugation (7,000×*g*, 30 min, 4°C) and filtered through a 0.22 µm Millipore filter to remove any remaining bacteria. Supernatants were concentrated to 30X using Amicon Ultra-15 30 K (Millipore, Billerica, USA), then centrifuged at 27,000×*g* for 1 h at 4°C to remove cellular debris and finally sterilized by filtration through a Millipore 0.22 µm filter.

### Phenotype characterization

#### Colony characterization

*P. aeruginosa* VR1 and VR2 isolates were grown on Luria Bertani (LB) agar plates for 24–48 h at 37°C and colony morphotypes were visually inspected for color, shape, edges regularity, and mucoidy.

#### Motility assays

Swimming plates (1 % tryptone, 0.5 % NaCl and 0.3 % (w/v) agar) were point-inoculated from an overnight culture with a sterile toothpick and incubated at 37°C for 24 h. The zone diameter was measured to assess swimming motility.

Swarming plates (0.5% peptone, 0.3% yeast extract, 0.5% NaCl, 0.5% d-glucose and 0.5% (w/v) agar) were point-inoculated from an overnight culture with a sterile toothpick and incubated at 37°C for 24 h. Swarming motility was assessed by measuring the circular turbid zones formed by the bacterial cells migrating away from the point of inoculation.

Twiching motility was evaluated on LB 1% (w/v) agar plates. Overnight cultures were stabbed with a sterile toothpick through the agar layer to the bottom of the Petri dish. The plates were then incubated at 37°C for 48 h. Twiching motility was examined by measuring the diameter of the halo formed in the plastic-agar interface.

#### Biofilm formation assay

Bacterial cells were grown at 37°C in TSB-1% glucose until they reached the exponentially growing phase (OD_650nm_ = 0.4). Exponentially growing cells were then diluted in TSB-1% glucose medium to reach 10^6^ CFU/mL. Two hundred microliter of each cell suspension were used to inoculate sterile flat-bottomed polystyrene microtiter plates (CytoOne, Starlab) and plates were incubated aerobically at 37°C without agitation for 48 h to allow biofilm formation. After incubation, the planktonic cells were aseptically aspirated, washed with sterile physiological solution and dried. For biofilm quantification, 100 µL of 1% methylene blue were added to each well and the plate maintained for 15 min at room temperature. The wells were subsequently slowly washed once with sterile water and dried at 37°C. The methylene blue bound to the biofilm was extract using 100 µL of 70% ethanol and the absorbance measured at 570 nm using “A3 Plate Reader” microplate reader (DAS Srl, Italy). All the mentioned experiment were performed in triplicate.

### Virulence factor assays

The presence of virulence factors in culture supernatants was evaluated after growth of the bacterial strains with and without AZM.

#### Gelatin-zymography for analysis of the protease profile

We used gelatin-zymography to investigate the presence of total metalloprotease activity, by visualization of clear bands (areas of gelatin digestion) over a deep blue background after Coomassie staining. Five millilitres of 5X SDS sample buffer (5% SDS, 0.5 M Tris–HCl pH 6.8, 25% glycerol) were added to 20 µL of culture supernatants. The sample was run on a SDS-PAGE gel containing 1 mg/mL gelatin (Sigma-Aldrich) as described [12]. In order to compare metalloproteases expression/release by the different strains, for each sample we loaded equal aliquots of culture supernatants normalized to a 0.2 final OD after overnight growth starting from 0.1 OD. The gel was washed twice (20 min per cycle) with 2.5% Triton X-100 at room temperature, then incubated in 200 mL of activation buffer (10 mM Tris–HCl, 1.25% Triton X-100, 5 mM CaCl_2_, 1 mM ZnCl_2_) overnight at 37°C and finally stained with Coomassie Brilliant Blue G-250 in 20% methanol/10% phosphoric acid/10% ammonium sulfate and destained in water. Gel images were captured by ImageQuant LAS 4000 (GE Healthcare Life Sciences, Milan, Italy).

#### Western blot analysis of alkaline metalloprotease AprA

Proteins were precipitated from 12 mL of culture supernatants by addition of 10% (final concentration) trichloroacetic acid with stirring at 4°C. The sample was then centrifuged at 3,000*g* for 30 min and washed 3 times with an excess of acetone:methanol (8:1). The pellet was air-dried, resuspended in SDS sample buffer, subjected to SDS-PAGE and Western blotting as described [12]. Briefly, protein bands were transferred from the 10% acrylamide gel onto a nitrocellulose membrane (HybondTM ECLTM, Amersham, NJ, USA), using a mini trans-blot apparatus (Bio-Rad, California, USA) following the manufacturer’s instructions. The membrane was incubated with a 1:500 dilution of purified rabbit IgG against *P. aeruginosa* alkaline protease (AprA), a kind gift from G. Döring (University of Tübingen, Germany), in TBS-T with 1% BSA overnight at 4°C. The blot was washed four times in TBS-T and then incubated for 1 h at room temperature with donkey anti-rabbit IgG secondary antibody conjugated to horseradish peroxidase (Amersham, NJ, USA) diluted 1:15,000 in TBS-T with 1% BSA. Bound proteins were visualized using the ECL detection system (Millipore Corporation, Bedford, MA, USA).

#### In vitro imaging probe

A near infrared (NIR)-activatable “smart” probe (Perkin Elmer, Inc. Boston, MA, USA) was used for imaging protease activities. Specifically, MMPsense 750 FAST is a proteases activatable fluorescent in vivo imaging agent that is activated by key disease associated-proteinases such as MMPs including MMP-2, -3, -9 and -13 and bacterial proteases. Smart probes are optically silent in their inactivated state and becomes highly fluorescent following protease-mediated activation.

The MMPsense 750 FAST probe was added to 50 μL of culture supernatants at the final concentration of 0.02 nmoles in a 96-well plate and an imaging system (IVIS, Caliper Life Sciences, Alameda, CA, USA) was used to quantify the time course of the fluorescence every 30 s from 1 to 40 min.

#### Pyoverdine assay

The production of pyoverdine by *P. aeruginosa* was measured spectrophotometrically by a modification of a standard method. Overnight cultures of the strains were diluted to an optical density at 600 nm (OD_600_) of 0.1 in 30 mL of King’s B broth (low-iron medium) with and without 8 µg/mL AZM and grown at 37°C until they reached an OD_600_ of ≈2–3 (after 16 h). Cultures were normalized to OD_600_ of 0.2 in order to reproduce the conditions of supernatants used for mice stimulation. The absorbance of cell-free supernatants was measured at 405 nm. The concentration of pyoverdine was calculated by using the extinction coefficient (1.9 × 10^−4^ M^−1^ cm^−1^).

#### Pyocyanin assay

The pyocyanin assay is based on the absorbance of pyocyanin at 520 nm in acidic solution (Essar et al. 1990). Bacterial cultures were grown in LB medium with or without AZM following the same conditions described for pyoverdine assay. Five milliliters of cell-free supernatants were extracted with 3 mL of chloroform. The lower phase was mixed with 1 mL of 0.2 M HCl, and the absorbance of the resulting upper pink phase was measured at 520 nm (*A*_520_). Concentration, expressed as μg of pyocyanin per millilitre of culture supernatant, was determined by multiplying the A520 value by 17.072.

### Experimental animals

Female inbred BalbC (7–8 week-old) mice were purchased from Harlan Laboratories Italy (San Pietro al Natisone, Udine, Italy). Prior to use, animals were acclimatized for at least 5 days to the local vivarium conditions (room temperature: 20–24°C; relative humidity: 40–70%; 12-h light–dark cycle), having free access to standard rodent chow and softened tap water. Animal experiments were conducted in compliance with national (Decreto Legislativo numero 26, 4 Marzo 2014) and international laws and policies (Guide for the Care and Use of Laboratory Animals) [17]. Animal studies were approved by the Institutional Animal Care and Use Committee at Chiesi Farmaceutici, Parma, Italy.

### In vivo gene delivery

The bIL-8-Luc plasmid (Department of Medical Veterinary Science, University of Parma, Italy) was obtained by sub-cloning the 2,030 bp IL8 bovine promoter, amplified by PCR from Madin–Darby bovine kidney (MDBK; ATCC #CCL-22) genomic DNA and sub-cloned into the digested pGL3basic vector (Promega) as previously described [8]. We applied in vivo JetPEI (Polyplus Transfection) as a carrier for delivering DNA to lung tissues. The DNA and JetPEI mix was formulated according to the product manual with a final N/P ratio of 7. Briefly, 40 μg of bIL-8–luc and 7 μL of JetPEI were both diluted into 200 μL 5% glucose. The two solutions were then mixed and incubated for 15 min at room temperature. The entire mixture was injected intravenously into BalbC mice and the expression of bIL-8–Luc was monitored through imaging with an IVIS imaging system.

### In vivo bioluminescence imaging

Transfection per se causes a mild lung inflammatory response and bIL-8 activation that is detectable by bioluminescence imaging (BLI) up to 3–4 days after DNA injection and disappears completely after 1 week [11].

One week after DNA delivery, the transient transgenic mice were injected intraperitoneally (i.p.) with luciferin (150 mg/kg) and BLI was recorded in order to check the baseline activation of the IL-8 pathway. Briefly, 10 and 15 min following luciferin injection, mice were lightly anesthetized with isoflurane and images were obtained using the IVIS imaging system: an average of photons emitted from the chest of the mice was quantified using Living Image^®^ software (Caliper Life Sciences, Alameda, CA, USA). The following day, mice were intratracheally challenged with 10X supernatants and BLI was recorded at 4, 24, and 48 h. Data were expressed as mean folds of induction (FOI) over the baseline activation of each mouse.

### Bronchoalveolar lavage and cytokine

Twenty-four hours after intratracheal challenge, animals were weighted, anaesthetized with isoflurane and sacrificed by bleeding from the abdominal aorta for bronchoalveolar lavage fluid (BALF) collection, performed as previously described [8]. BALF supernatants were frozen at −80°C for simultaneous quantitation of multiple cytokines/chemokines using a Bio-Plex™ Cytokine Assay Kit (Bio-Rad Laboratories, Segrate, Milano, Italy).

The cell pellet was suspended in 0.2 mL of PBS and total and differential cell counts were obtained using an automated cell counter (Dasit XT 1800J, Cornaredo; Milano, Italy).

### Reagents

In vivo JetPEI DNA transfection reagent (Polyplus Transfection) was obtained from Euroclone (Milan, Italy), d-luciferin was obtained from Perkin Elmer Inc. (Boston, MA, USA). AZM was from Pfizer (Italy).

### Data analysis

Experimental values were expressed as the mean and standard error of the mean (SEM). Statistical analysis was performed using one-way analysis of variance followed by Dunnett’s t test (* p < 0.05, ** p < 0.01).

## Results

### Phenotypic characterization of *Pseudomonas aeruginosa* clinical isolates

We have previously characterized by large-scale proteomic analysis the proteins released by different *P. aeruginosa* strains and have demonstrated their different capability to induce pro-inflammatory factors in bronchial epithelial cells [12]. For this study, we selected two *P. aeruginosa* strains, VR1 and VR2, isolated from two patients affected by CF and showing significant differences as regards phenotypic characteristics and release of various established virulence factors. The phenotypic characterization of the two strains is shown in Fig. 1. VR1 and VR2 differentiate for the production of pyoverdin and pyocyanin which are released only in the VR1 supernatant, the capability of moving by swimming, swarming and twitching detectable in VR1 but non in VR2, the production of biofilm which is more evident in VR1 with respect to VR2 and by the level of protease activity, well detectable in the VR1 supernatant and very reduced in the VR2 one.

**Fig. 2:**
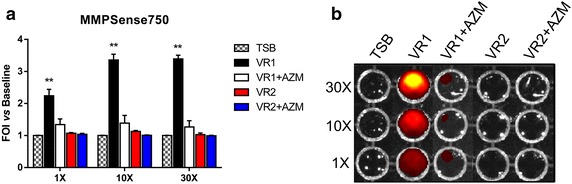
Protease activity of cell-free supernatants from *Pseudomonas aeruginosa* grown in the presence and the absence of azithromycin. MMPsense 750 FAST was added to supernatants from VR1 and VR2 strains grown in presence or absence of AZM (SnVR1 ± AZM and SnVR2 ± AZM) concentrated 1X, 10X and 30X. IVIS imaging system has been used to quantify the time course of the fluorescence. Data were expressed as induction folds over baseline (bacterial growth medium TSB). Results are reported as mean ± SEM and significance attributed when P < 0.05 (*) or P < 0.01 (**).

**Fig. 3:**
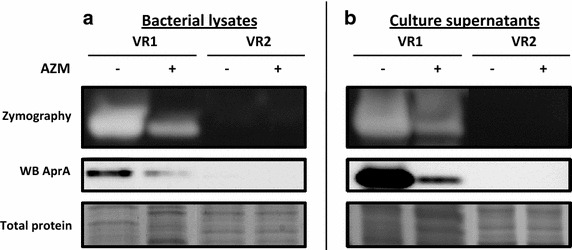
Analysis of metalloproteases activity and synthesis in VR1 and VR2 bacterial lysates and culture supernatants. MPs activity and synthesis are downregulated in VR1 + AZM and absent in VR2, both in lysates (**a**) and supernatants (**b**), as shown by zymography and western blot specific for AprA.

Culture supernatants were prepared from the bacterial strains by growing them in a culture medium in the absence (SnVR1 and SnVR2) and in the presence of a sub-MIC dose of AZM (SnVR1 + AZM and SnVR2 + AZM). The inhibitory action of AZM on the expression of virulence factors is clearly shown in Fig. 1. Culture supernatants were tested for the presence of proteolytic activity by a proteases activatable fluorescent probe (Fig. 2) and by zymography (Fig. 3b). The specific inhibitory effect of AZM on metalloprotease synthesis used as a marker of the efficacy of the treatment was also demonstrated by testing cell lysate and supernatants for the presence of proteolytic activity with a proteases activable fluorescent probe (Fig. 2) and by zymography and western blotting specific for the *P. aeruginosa* metalloprotease, AprA (Fig. 3).

### In vivo monitoring of lung inflammation induced by *P. aeruginosa* culture supernatants in IL-8 transiently transgenic mice

**Fig. 4:**
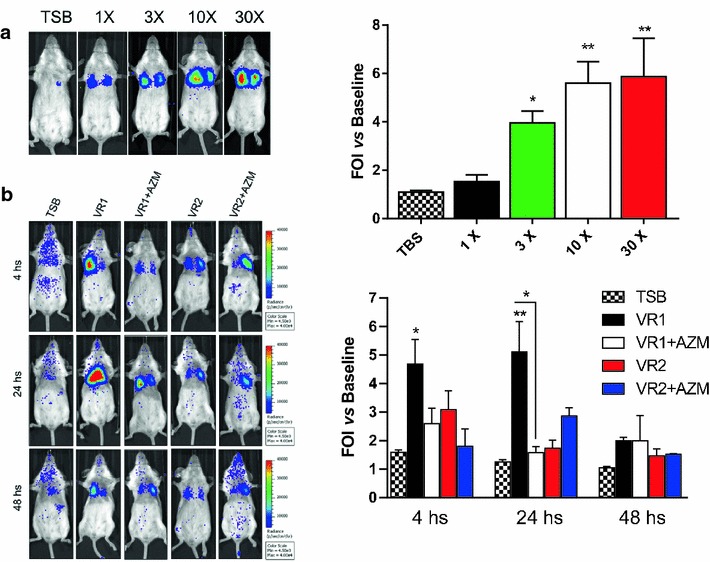
In vivo imaging of lung inflammation induced by *P. aeruginosa* culture supernatants on IL-8 transiently transgenic mice. **a** Representative images of mice (n = 3 per group) transiently transgenized with bIL-8-Luc and intratracheally instilled with bacterial cell-free 1X, 3X, 10X and 30X supernatants from VR1. The growth medium TSB was used as a control. Mice were monitored at 4, 24 and 48 h post stimulation by BLI drawing a region of interest (ROI) over the chest. **b** Representative images of mice (n = 8 per group) transiently transgenized with bIL-8-Luc and intratracheally instilled with bacterial cell-free, 10X supernatants from VR1 and VR2 strains grown in presence or absence of AZM (VR1 ± AZM and VR2 ± AZM). The growth medium TSB was used as a control. Mice were monitored at 4, 24 and 48 h post stimulation by BLI drawing a region of interest (ROI) over the chest. Data are also presented as light intensity quantification of the ROI using the LivingImage software. The experiment was repeated 3 times and each point represents the mean ± standard error of 8 animals. Data were expressed as FOI over baseline activity of each mice and statistical differences were tested by one way ANOVA followed by Dunnett’s post hoc test for group comparisons. Results are reported as mean ± SEM and significance attributed when P < 0.05 (*) or P < 0.01 (**).

In vivo monitoring of lung inflammation after intratracheal challenge with *P. aeruginosa* SnVR1 and SnVR2 at 1X, 3X, 10X and 30X concentrations was carried out by in vivo imaging in bIL-8 luc transient transgenic mice. Administration of the 10X SN concentration was sufficient to induce the maximal increase in BLI signal. In fact, the use of the 30X Sn preparation did not translate into a higher inflammation signal, indicating a saturation of the system at lower concentration (10X) (Fig. 4a). For this reason, further experiments were conducted using 10X concentration.

SnVR1 pro-inflammatory activity was clearly detectable as soon as 4 h post instillation, although the BLI signal reached the highest peak at 24 h (FOI 5.11 ± 1.07) and was still detectable at 48 h even if there is no statistical difference among the different groups (Fig. 4b). On the contrary, the SnVR2 did not induce detectable inflammation in the mouse lung at any time point. The BLI signal induced by the challenge with SnVR1 + AZI was significantly lower (FOI 1.58 ± 0.21) after 24 h with respect to the response induced by SnVR1 obtained from the same bacterial strain grown without the antibiotic (Fig. 4b).

### Recruitment of inflammatory cells and cytokine activation induced by *P. aeruginosa* culture supernatants

**Fig. 5:**
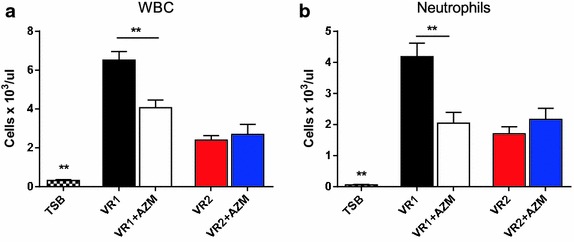
Immune cell recruitment during lung inflammation caused by *P. aeruginosa* supernatants. Cellular infiltration into the lung of mice intratracheally instilled with bacterial cell-free 10X supernatants from VR1 and VR2 strains grown in presence or absence of AZM (VR1 ± AZM and VR2 ± AZM). TSB is the bacterial growth medium and was used as a control. The amount of white blood cells and neutrophils found in BALF was expressed as number of cells per μl at 24 h post treatment. The experiment was repeated 3 times and each point represents the mean ± standard error of 8 animals. Results are reported as mean ± SEM and significance attributed when P < 0.05 (*) or P < 0.01 (**).

**Fig. 6:**
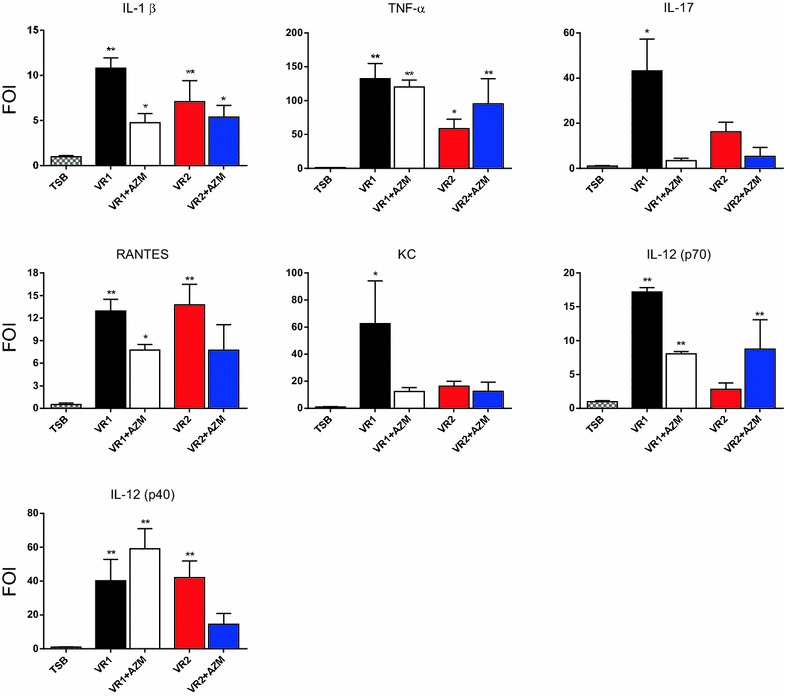
Cytokines concentration in mice BALF after stimulation with *P. aeruginosa* culture supernatants. Cytokine levels in BALF of bIL-8 transgenized mice 24 h after intratracheally challenge with bacterial cell-free supernatants from VR1 and VR2 strains grown in presence or absence of AZM (VR1 ± AZM and VR2 ± AZM). Data were expressed as FOI over the control (bacterial growth medium TSB) The experiment was repeated 3 times and each point represents the mean ± standard deviation of 8 animals. Results are reported as mean ± SEM and significance attributed when P < 0.05 (*) or P < 0.01 (**).

Twenty-four hours after mice stimulation with *P. aeruginosa* products, BALF was recovered from bIL-8 transgenic animals treated with both VR1 and VR2 supernatants in order to compare their effect on cell recruitment and cytokine expression. SnVR1, containing a series of important virulence factors, significantly stimulated total white blood cells (WBC) and neutrophils recruitment (respectively 6.52 × 10^3^ ± 0.44 and 4.19 × 10^3^ ± 0.43 cells/µL) and the expression of cytokines IL-1β, TNF-α, IL-17, RANTES, KC, IL 12 (p70) and IL 12 (p40) (Figs. 5, 6). Inflammatory cells (WBC 2.39 ± 0.23 × 10^3^ and neutrophils recruitment 1.70 ± 0.0.22 × 10^3^ cells/µL) and expression of the cited cytokines were lower in SnVR2 with respect to SnVR1. SnVR2 showed a comparable effect on the release of RANTES and IL 12 (p40).

BALF was recovered also from bIL-8 transgenic mice stimulated with SnVR1 + AZM and SnVR2 + AZM in order to evaluate the effect of the antibiotic on the pro-inflammatory activity of both preparations. The challenge with SnVR1 + AZM stimulated at a significant lower level WBC and neutrophils recruitment (respectively 4.07 ± 0.39 × 10^3^ and 2.05 ± 03.4 × 10^3^ cells/µL) (Fig. 5) and the expression of the cytokines IL-1β, IL-17, RANTES, KC and IL-12 (p70) but not that of TNF-α and IL 12 (p40) in comparison with animals treated with SnVR1 (Fig. 6). A significant difference between the stimulation caused by SnVR2 and SnVR2 + AZM was not observed except for RANTES and IL12-(p40) level (Fig. 6).

## Conclusions

The mechanisms and mediators that drive the induction and progression of chronic inflammation and alter lung function in a series of human diseases are not completely understood and this has severely hampered the development of effective treatments [1–3]. Although most of the animal models do not exactly mimic human lung diseases and each model has its own benefits and deficiencies, animal models that accurately reflect human disease pathophysiology continue to be essential for the understanding of pathogenic aspects as well as the development and validation of new therapies. In most mammalian models, TNFα, IL-1β, and IL-8 are central components of a complex cytokine network that initiates, amplifies, and sustains the inflammatory response in tissue [18]. Available evidence also supports the importance of this network in coordinating acute inflammatory responses within the lung. The mouse model used in this study, transiently transgenized with a luciferase reporter gene under the control of the IL-8 bovine promoter, can be easily employed for modeling and monitoring human lung inflammation due to the high similarity existing between respiratory diseases in ruminants and humans without the high costs and demands in terms of maintenance of ruminant models [8].

In the present study, the IL8/luciferase mouse model has been applied to the in vivo monitoring of lung inflammation induced by virulence factors released by *Pseudomonas aeruginosa* and to the evaluation of the anti-inflammatory action of AZM as an inhibitor of the synthesis of bacterial factors involved in pathogenicity. Data here presented show that the *P. aeruginosa* clinical strain VR1, isolated from early lung colonization in a CF patient, synthesizes flagella and biofilm, produces and releases pyocyanin and pyoverdine and proteins with metallo-protease activity. The synthesis and release of these virulence factors significantly decreases when bacteria were grown in the presence of AZM. The pro-inflammatory effect of strain VR1 has been shown in IL-8/luciferase transiently transgenic mice by applying in vivo imaging and further confirmed by the increase in WBC and neutrophils recruitment and cytokine levels in the airways of transgenized mice. On the contrary, the supernatant from bacteria grown in the presence of AZM, a condition associated with a much lower production of virulence factors, stimulated the inflammatory response only at a very reduced level. Similar low levels of IL-8 promoter activation was observed when the supernatant of the *P. aeruginosa* VR2 strain, lacking the specific virulence factors, was instilled in the airways of IL-8/luciferase transgenized mice.

These data support the notion that the clinical benefits associated to AZM treatment in CF patients might be at least in part due to the lowering of the exoproduct synthesis induced by the antibiotic in bacterial cells.

Data obtained in this study demonstrate that the model here described is suitable to non-invasive real time monitoring of lung inflammatory response to bacterial products and to confirm and better understand the mechanism of action of AZM (or any other compound of interest), an antibiotic frequently used in the therapy of CF. The non invasive nature of this mouse model and the possibility for bIL-8-luc transiently transgenized mice to be stimulated with bacterial products for long times [8] enables the monitoring of a biological process longitudinally in the same mouse and represents an obvious advance for functional as well as pharmacological studies. The model might be adapted and applied to study the pathogenesis of lung inflammatory diseases such as CF, to identify bacterial/nonbacterial factors with pro-inflammatory activity and to predict the possible therapeutic effect of known and new molecules with a presumptive anti-inflammatory action.

## Abbreviations

AZM: azithromycin
NIR: near infrared
BLI: bioluminescence imaging
BALFs: bronchoalveolar lavage fluids
